# *Emericella* *quadrilineata* as Cause of Invasive Aspergillosis

**DOI:** 10.3201/eid1404.071157

**Published:** 2008-04

**Authors:** Paul E. Verweij, János Varga, Jos Houbraken, Antonius J.M.M. Rijs, Frans M. VerduynLunel, Nicole M.A. Blijlevens, Yvonne R. Shea, Steven M. Holland, Adilia Warris, Willem J. G. Melchers, Robert A. Samson

**Affiliations:** * Radboud University Nijmegen Medical Center, Nijmegen, the Netherlands; † Centraal Bureau voor Schimmelcultures Fungal Biodiversity Centre, Utrecht, the Netherlands,; ‡ University of Szeged, Szeged, Hungary; § National Institutes of Health, Bethesda, Maryland, USA

**Keywords:** Aspergillosis, Emericella, Laboratory diagnosis, research

## Abstract

We noted a cluster of 4 cases of infection or colonization by *Emericella* spp., identified by sequence-based analysis as *E. quadrilineata*. Sequence-based analysis of an international collection of 33 *Emericella* isolates identified 12 as *E. nidulans*, all 12 of which had previously been identified by morphologic methods as *E. nidulans*. For 12 isolates classified as *E. quadrilineata*, only 6 had been previously identified accordingly. *E. nidulans* was less susceptible than *E. quadrilineata* to amphotericin B (median MICs 2.5 and 0.5 mg/L, respectively, p<0.05); *E. quadrilineata* was less susceptible than *E. nidulans* to caspofungin (median MICs, 1.83 and 0.32 mg/L, respectively, p<0.05). These data indicate that sequence-based identification is more accurate than morphologic examination for identifying *Emericella* spp. and that correct species demarcation and in vitro susceptibility testing may affect patient management.

## Abstract 2

This opportunistic fungus is frequently misidentified because of its morphologic similarity to *E. nidulans*.

The genus *Aspergillus* includes >250 species; ≈20 have been reported to cause opportunistic infections in humans. The most important human pathogens in this genus are *A. fumigatus, A. flavus, A. niger, A. terreus,* and *Emericella* *nidulans* (anamorph: *A. nidulans*) (*1*). *A. fumigatus* is the most common cause of invasive aspergillosis, a condition associated with substantial severity and mortality rates (*2*). Invasive infections caused by *E. nidulans* are uncommon in animals and humans (*3*–*5*); in humans they appear to occur predominantly in patients who have chronic granulomatous disease (CGD), a rare disorder of phagocytes in which the absence of both superoxide and hydrogen peroxide production in phagocytes predisposes patients to bacterial and fungal infections. Invasive *E. nidulans* infections in this patient group are associated with higher mortality rates than those caused by *A. fumigatus* (*6*,*7*). The most common site of infection is the lungs; other manifestations are subcutaneous abscesses or liver abscesses, suppurative adenitis, osteomyelitis, fungemia, cellulitis, and meningitis (*7*,*8*). Within the genus *Emericella*, other species have only rarely been identified as agents of human or animal infections.

The identification of *E. nidulans* in clinical microbiology laboratories is commonly based on the characteristic microscopic morphology, the production of hülle cells, or the production of ascospores. *A. fumigatus* is identified by its heat tolerance; other species fail to grow when incubated at high temperature, typically 48°C.

We recently noted a cluster of infection or colonization by *E. quadrilineata*, a species closely related to *E. nidulans*. Within a 3-month period, 4 cases were identified at the Radboud University Nijmegen Medical Center. No apparent epidemiologic link between the cases was found because each patient was cared for in a different ward, and 2 patients with invasive aspergillosis were admitted directly from home. One of the latter 2 patients was a 10-year-old boy with X-linked CGD and a probable diagnosis of invasive pulmonary aspergillosis (*9*); the other patient was a 60-year-old man who had chronic lymphocytic leukemia and in whom cerebral aspergillosis later developed and was confirmed by biopsy. From the other 2 patients, who had no signs and symptoms of invasive fungal disease, *E. quadrilineata* was cultured from respiratory specimens. No laboratory contamination was evident during the period in which the cluster occurred. No subsequent cases were identified. Morphologic species identification was difficult, and we had to rely on sequence-based identification, which prompted this investigation of the role of *E. quadrilineata* as a causative agent of invasive aspergillosis.

## Methods

### Data and Strain Collection

We searched the PubMed literature for cases of infections caused by *E. quadrilineata* (anamorph: *Aspergillus tetrazonus*) or *E. nidulans;* search terms were *Emericella*, *quadrilineata, tetrazonus, nidulans*, and aspergillosis. For those articles that described infections by *E. quadrilineata* or *E. nidulans,* we asked the authors to send us their isolates for sequence analysis. We also approached colleagues who care for patients with CGD or might otherwise have a collection of *E. nidulans* isolates. We also searched our department’s fungal culture collection for *E. nidulans* isolates. It is our policy to store all *Aspergillus* isolates cultured from clinical specimens sent to our laboratory, regardless of the clinical relevance of the isolate. Finally, we added *E. nidulans* and *E. quadrilineata* isolates deposited in the culture collection of the Centraal Bureau voor Schimmelcultures ([CBS], Utrecht, the Netherlands). The final collection totaled 33 *Emericella* isolates, with 11 isolates from the CBS culture collection (type strains *E. quadrilineata, E. nidulans, E. nidulans* var. *echinulata*) and 1 isolate from the National Collection of Pathogenic Fungi. Ten isolates were from our own culture collection (including the 4 encountered in the cluster of cases), and 11 isolates were from 5 other medical centers; some of these isolates had been cultured as causes of infection and previously reported (online Appendix, available from www.cdc.gov/EID/content/14/4/566-appT.htm) (*8*,*9**–**12*). Seven isolates had been cultured from patients with confirmed invasive aspergillosis, and 2 were from patients with probable cases (online Appendix, available from www.cdc.gov/EID/content/14/4/566-appT.htm).

### Morphologic Identification

*Aspergillus* isolates are routinely identified by their macroscopic colony morphology and the microscopic morphology of their anamorphic features. When teleomorph features were also used to identify an isolate, teleomorph nomenclature, such as *Emericella* spp., was used to report the strain. In addition, the isolates were incubated at 48ºC, which precludes the growth of most *Aspergillus* spp. except *A. fumigatus*.

### Sequence-based Identification

Sequence-based identification in the routine clinical microbiology laboratory was carried out by sequencing of parts of the internal transcribed spacer (ITS) 1 and 2 regions. Total DNA of the *Emericella* cultures was extracted by using the MagNa Pure Total NA isolation kit (Roche Diagnostics Nederland BV, Almere, the Netherlands). Then the ITS 1 and 2 sequence was amplified by PCR with primers ITS1 (5′-TCCGTAGGTGAACCTGCGG-3′) and ITS4 (5′-TCCTCCGCTTATTGATATGC-3′) as described (*13*). After purification, the PCR product was sequenced with the BigDye Terminator v3 kit (Applied Biosystems, Foster City, CA, USA).

In addition to the above-mentioned sequenced-based identification, parts of the β-tubulin and calmodulin genes were sequenced. The *Emericella* cultures were cultivated in 2 mL malt peptone broth by using 10% (vol/vol) of malt extract (Oxoid, Basingstoke, UK) and 0.1% (wt/vol) bacto peptone (Difco, Becton Dickinson, Le Pont de Claix, France). The cultures were incubated at 25°C for 7 days. DNA was extracted from the cells by using the Masterpure yeast DNA purification kit (Epicentre Biotechnologies, Madison, WI, USA) according to the manufacturer’s instructions. Amplification of part of the β-tubulin gene was performed by using the primers Bt2a and Bt2b (*14**,**15*). Amplifications of the partial calmodulin gene were set up as described (*16*). Sequence analysis was performed with the BigDye Terminator Cycle Sequencing Ready Reaction Kit for both strands. Sequences were aligned by using ClustalX software (*17*) and were improved manually.

Evolutionary distances between the sequences were calculated by using the Kimura formula (*18*) and DNADIST program of the PHYLIP program package (*19*). Phylogenetic trees were prepared by using the neighbor-joining method (*20*) and the NEIGHBOR program of the PHYLIP package. Bootstrap values were calculated from 1,000 replications of the bootstrap procedure by using programs SEQBOOT, DNADIST, NEIGHBOR, and CONSENSE of the PHYLIP package (*19**,**21*). For parsimony analysis, PAUP* version 4.0 software was used (*22*). *E. heterothallica* was used as an outgroup in these experiments. The unique β-tubulin and calmodulin sequences were deposited in the GenBank nucleotide sequence database under accession nos. EF591677–EF591702.

### Antifungal-Drug Susceptibility Testing

Antifungal-drug susceptibility testing of *Emericella* isolates was performed by using a microbroth dilution assay, as described by the Clinical Laboratory Standards Institute (M38-A) for amphotericin B (Bristol-Myers Squibb, Woerden, the Netherlands), itraconazole (Janssen-Cilag, Beerse, Belgium), voriconazole (Pfizer, Capelle aan den IJssel, the Netherlands), posaconazole (Schering-Plough, Maarsen, the Netherlands), caspofungin (MSD, Haarlem, the Netherlands), and terbinafine (Novartis, Arnhem, the Netherlands) (*23*). MICs were determined for all drugs except caspofungin, for which a microscopic endpoint was used (minimum effective concentration) (*24*). All in vitro susceptibility testing was performed in duplicate.

### Statistical Analysis

After being transformed logarithmically, MIC dilutions were compared by using the Mann-Whitney U test. Data on growth at different antifungal drug concentrations were normalized by setting the corrected optical density of the growth control at 0% and the lowest optical density at 100%. Growth characteristics were analyzed by nonlinear regression analysis that used a 4-parameter logistic model and created a sigmoidal curve. Test runs determined deviation of the model, and goodness-of-fit was tested by determining r^2^ values. In addition to comparing MICs, we determined the drug concentration at which growth was 50% that of the control (50% maximal effective concentration [EC_50_]) and calculated and fitted the slope of the curve (GraphPad Prism, San Diego, CA, USA). For all drugs except caspofungin, the EC_50_ values and slopes were compared for *E. nidulans* and *E. quadrilineata*.

## Results

### Species Identification

The 4 isolates from the cluster of cases grew on Sabouraud-dextrose agar as velvety, brownish-green colonies with a purplish reverse side. Conidiophores were light brown with hemispherical vesicles bearing metulae and biseriate philalides on the upper half. Conidia were spherical, smooth walled, subhyaline, finely roughened, and 3–4 μm in diameter. After ≈3 weeks of incubation, purple ascocarps formed, surrounded by characteristic hülle cells. Asci were spherical, 8 spored, 10–13 μm in diameter, and evanescent. The ascospores were reddish purple, lenticular, 5–6 × 3–4 μm, and smooth. The morphologic features were consistent with *E. nidulans.* As part of the diagnostic process, the isolates were incubated at 48ºC, and all isolates showed some growth, which was considered inconsistent with *E. nidulans*. Because of this discrepancy, sequence-based identification was performed. However, all 33 isolates from the subgenus *Nidulantes* section that were analyzed in this study grew at 48°C, which indicates that incubation at this temperature does not fully distinguish between this section and *A. fumigatus*.

### Sequence-based Analysis

The ITS sequence analysis of the 4 isolates was consistent with that of *E. quadrilineata*, although there were only 1 or 2 mismatches with the base-pair sequence of *E. nidulans*. The morphologic features of *E. nidulans* and *E. quadrilineata* are very similar; only the microscopic shape of the ascospores shows subtle differences. Ascospores of *E. nidulans* have 2 longitudinal crests, as opposed to *E. quadrilineata,* which has 4 short equatorial crests. The resolution of the ITS region was considered too low to unambiguously differentiate between *E. nidulans* and *E. quadrilineata*, and further sequence-based identification was performed at CBS by using partial β-tubulin and calmodulin sequence data.

**Figure 1:**
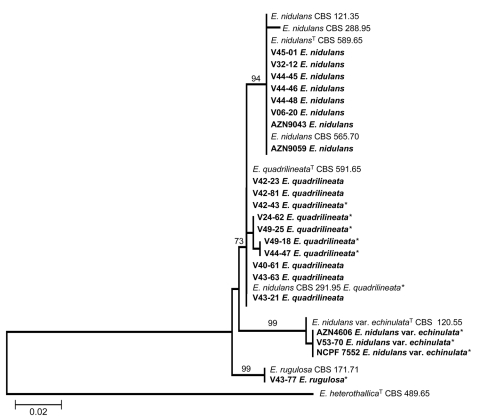
Neighbor-joining tree based on β-tubulin sequence data of the *Emericella* isolates examined. Clinical isolates are set in **boldface**. Numbers above branches are bootstrap values. Only values >70% are indicated. ^T^ indicates the type strain; * indicates the isolates that had been misidentified by morphologic identification as *E. nidulans*. Scale bar represents genetic distance calculated by the Kimura 2-parameter model (*18*).

**Figure 2:**
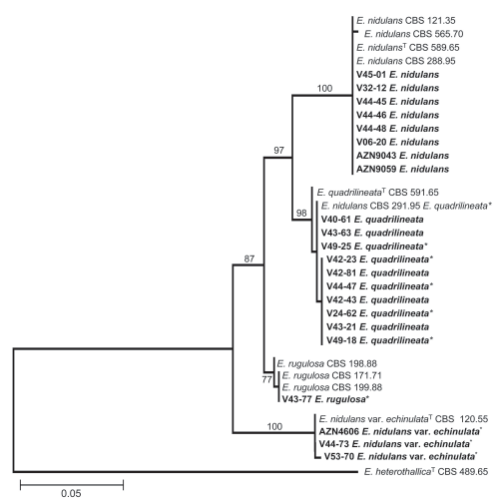
Neighbor-joining tree based on calmodulin sequence data of *Emericella* isolates examined. Clinical isolates are set in **boldface**. Numbers above branches are bootstrap values. Only values >70% are indicated. ^T^ indicates the type strain; * indicates the isolates that had been misidentified by morphologic identification as *E. nidulans*. Scale bar represents genetic distance calculated by the Kimura 2-parameter model (*18*).

During analysis of part of partial β-tubulin gene sequences, we analyzed 367 bases of all 33 isolates. Among the polymorphic sites, 23 were phylogenetically informative. The neighbor-joining tree (Figure 1) based on partial β-tubulin gene sequences had the same topologic features as 1 of the 2 maximum-parsimony trees constructed by the PAUP program (length 94 steps, consistency index 0.9787, retention index 0.9762). The calmodulin dataset included 489 bases, with 50 parsimony informative sites. The topologic features of the neighbor-joining tree (Figure 2) and 1 of the 2 most parsimonious trees were the same (tree length 162, consistency index 0.9691, retention index 0.9854). Molecular data indicated that 12 of 33 isolates could be classified as *E. nidulans*, all of which had previously been identified as *E. nidulans* by microscopic examination of morphologic characteristics or other methods. For the 12 isolates classified as *E. quadrilineata*, only 6 had previously been identified accordingly. These 6 isolates included the 4 in our cluster, 1 from the CBS culture collection, and 1 previously reported as the cause of onychomycosis (*12*). The remaining 6 isolates had been previously identified as *E. nidulans* (online Appendix, available from www.cdc.gov/EID/content/14/4/566-appT.htm). Of these, 1 belonged to the CBS culture collection, 1 was reported as the cause of cerebral aspergillosis (*11*), and 2 were from patients with CGD and confirmed invasive aspergillosis.

**Figure 3:**
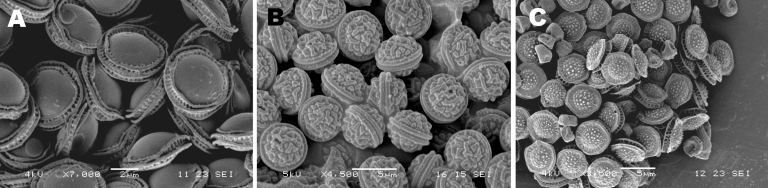
Scanning electron microscopic images of ascospores of some *Emericella* isolates. A) *E.* *quadrilineata* V43-63; B) *E. rugulosa* V43-77; C) *E. nidulans* var. *echinulata* 4606. Scale bars represent 5 μm.

A total of 4 isolates were classified as *E. rugulosa*, 1 of which had been previously reported as *E. nidulans* (*10*). A total of 4 isolates were identified as *E. nidulans* var. *echinulata*, 2 of which had caused invasive aspergillosis in patients with CGD and had been presumptively identified as *E. nidulans*. Scanning electron microscopy of the ascospores of some isolates supported their species assignment (JEOL 5600LV scanning electron microscope [JEOL, Tokyo, Japan] equipped with an Oxford CT1500 Cryostation [Oxford Instruments, Oxford, UK]) (Figure 3) (*25*).

### In Vitro Susceptibility

The in vitro activity of antifungal agents against the *Emericella* isolates is shown in the online Appendix, available from www.cdc.gov/EID/content/14/4/566-appT.htm. Overall, terbinafine was the most active drug in vitro, followed by posaconazole, which was the most active azole. For statistical comparisons, we used 12 *E. nidulans* and 12 *E. quadrilineata* isolates. By comparing MICs, statistically significant differences in drug activity were found for amphotericin B, voriconazole, and posaconazole (Table). These differences were also found when EC_50_ values and slopes were compared for both species (data not shown). Comparison of minimum effective concentrations showed caspofungin to be significantly more active against the *E. nidulans* isolates (p<0.05). Although only 4 *E. nidulans* var. *echinulata* isolates were analyzed, the susceptibility profile of these isolates was similar to that of *E. quadrilineata* (and not of *E. nidulans*); for amphotericin B, MICs were low, and for caspofungin, MICs were high.

**Table:** Antifungal activities against *Emericella* *nidulans* and *E. quadrilineata*

Drug	MIC, mean		Significance*
	*E. nidulans* (n = 12)	*E. quadrilineata* (n = 12)	
Amphotericin B	2.5	0.5	p<0.05
Itraconazole	0.07	0.13	NS
Voriconazole	0.26	0.39	p<0.05
Posaconazole	0.25	0.22	p<0.05
Caspofungin†	0.32	1.83	p<0.05
Terbinafine	0.01	0.009	NS

*NS, not significant. †Mean minimum effective concentration was compared.

### Literature Review

Three cases of infection due to *E. quadrilineata* have been documented. One patient was a 60-year-old man from northern India, who had a fingernail infection that affected all 5 nails of 1 hand. The strain was repeatedly cultured from 1 nail, and septate hyphal elements were seen in a portion of an excised nail. The patient was treated with itraconazole, but the response could not be evaluated (*12*). Invasive aspergillosis caused by *E. quadrilineata* has been described for 2 patients, both of whom had sinusitis. One of these, a 28-year-old woman, had acute nonlymphoblastic leukemia and had undergone allogeneic bone marrow transplantation. She developed sinusitis with orbital involvement 2 months after transplantation. The diagnosis was confirmed by biopsy, and the patient was successfully treated with a combination of surgical debridement, granulocyte transfusions, and intravenous amphotericin B–cholesterol sulfate colloidal dispersion (*26*). The other patient was a 28-year-old man who had received an allogeneic bone marrow transplant for acute myeloid leukemia. Left orbital swelling, facial pain, and nasal congestion developed 68 days after transplantation. *E. quadrilineata* was cultured from a biopsy specimen; the patient was successfully treated with external ethmoidectomy, granulocyte transfusions, and topical and systemic therapy with a lipid formulation of amphotericin B. The fungal infection resolved (*27*).

## Discussion

Until this report, 2 cases of invasive aspergillosis caused by *E. quadrilineata* had been described; each case had been reported as sinusitis in patients who had undergone bone marrow transplantation for hematologic malignancy. We add 1 case of central nervous system aspergillosis and 3 cases of invasive pulmonary aspergillosis in patients with CGD. The 3 cases may not be surprising because *E. quadrilineata* is very closely related to *E. nidulans*, a fungus known to cause infections in humans (*3*,*5*,*28**–**30*), primarily in patients with CGD (*6*,*7*). *Emericella* spp. other than *E. nidulans* are less frequently reported as causative agents of infectious disease. In addition to the above-mentioned human infections, *E. quadrilineata* has been identified as a causative agent of mycosis in animals (*31*). We have found only 1 report each of *E. rugulosa* (*4*) and *E. nidulans* var. *echinulata* (*32*) as the cause of human or animal infections.

Discriminating *E. nidulans* and *E. quadrilineata* by morphologic characteristics is virtually impossible. Only the ascospore ornamentation differs, and the subtle differences cannot be seen by using light microscopy. The most frequently used technique for their unambiguous identification is scanning electron microscopic examination of the lining of the ascospores (*25*). However, fruiting bodies are usually formed after a rather long incubation period (1–2 weeks). And although *E. nidulans* was found to form cleistothecia in the human body (*33*), clinical isolates often lose their ability to form sexual reproductive structures and ascospores (*34**,**35*). Given these difficulties, we anticipate that reliance on phenotypic characteristics alone would cause misidentification of *E. nidulans,* rather than correct identification of *E. quadrilineata,* as the cause of invasive aspergillosis. The sequence-based analysis showed that this was indeed the situation; 50% of the *E. quadrilineata* isolates had previously been identified as *E. nidulans*. Among these was a case of cerebral aspergillosis, the second case observed in our cluster of cases. Despite the close morphologic and genetic relatedness between *E. nidulans* and *E. quadrilineata*, the activity of antifungal agents differed significantly, which supports the conclusion that biological differences exist between these species. The triazoles were active in vitro; posaconazole showed the greatest activity, which is also observed for most *Aspergillus* spp. Although significant differences were found for activity of voriconazole and posaconazole, these differences appear to be not clinically relevant, given the small differences in MIC values (Table). However, for amphotericin B and caspofungin, the observed differences in activity may be clinically important. Amphotericin B was less active against *E. nidulans* than against *E. quadrilineata*. In vitro resistance of *E. nidulans* against amphotericin B has been recognized (*36*), although the testing method has been shown to substantially affect the activity found (*37*). However, lack of activity of amphotericin B has also been reported in experimental models of infection and in cases reported in the literature (*8*,*38*). Caspofungin was less active against *E. quadrilineata* than against *E. nidulans*. Caspofungin was shown to be effective against *E. nidulans* in a murine model of systemic infection (*38*), but no data are available for *E. quadrilineata.* Although the allylamine terbinafine is not used for treatment of patients with invasive aspergillosis, the drug is highly active against both *E. nidulans* and *E. quadrilineata*. The isolates were inhibited at concentrations as low as 0.015 mg/L.

Identification of molds primarily relies on morphologic criteria such as the macroscopic colony morphology and the microscopic morphology of the conidia and the structures bearing the conidia. Morphologic identification underestimates differences among species and among members of the same species. This was recently shown for the section *Fumigati*, in which *A. lentulus* and *A. udagawae* were among isolates phenotypically identified as *A. fumigatus* (*34**,**35*). We made a similar observation when 10 of 33 *Emericella* isolates were found to be misidentified. Correct species demarcation is important from a taxonomic viewpoint but can also have clinical relevance. Within the *Aspergillus* section of *Fumigati,* the newly identified species *A. lentulus* was shown to be more resistant than *A. fumigatus* to antifungal drugs (*35*). Therefore, correct species identification will affect the choice of antifungal therapy. Differences in drug activity were also apparent in the *Emericella* spp. we examined. Another important reason for correct species identification is the detection of outbreaks of infection, which could warrant interventions to prevent invasive fungal infection in immunocompromised persons or lead to epidemiologic surveys to identify sources of spread of fungal spores. However, the resolution of sequencing of the ITS region is too low to reliably differentiate between *E. nidulans* and *E. quadrilineata;* therefore, in vitro susceptibility testing might be appropriate in those laboratories that do not have access to sequencing of β-tubulin and calmodulin genes.

Molecular techniques in addition to morphologic identification have identified a role of *E. quadrilineata* as an opportunistic fungal pathogen, especially in patients with CGD and in those with hematologic malignancy. These molecular techniques will help identify and discriminate more accurately within the current fungal species and will give more insight into the pathogenesis of fungal infection.

## Supplementary Material

Appendix Table Chagas disease cases reported in France since 2004*

## References

[1] Denning DW. Invasive aspergillosis. Clin Infect Dis. 1998;26:781–805. 10.1086/513943 9564455

[2] Pagano L, Caira M, Picardi M, Candoni A, Melillo L, Fianchi L, Invasive aspergillosis in patients with acute leukemia: update on morbidity and mortality—SEIFEM-C report. Clin Infect Dis. 2007;44:1524–5. 10.1086/517849 17479958

[3] Joshi KR, Mathur DR, Sharma JC, Vyas MC, Sanghvi A. Mycetoma caused by *Aspergillus nidulans* in India. J Trop Med Hyg. 1985;88:41–4. 3894683

[4] Knudtson WU, Kirkbride CA. Fungi associated with bovine abortion in the northern plains states (USA). J Vet Diagn Invest. 1992;4:181–5. 1616983 10.1177/104063879200400211

[5] Lucas GM, Tucker P, Merz WG. Primary cutaneous *Aspergillus nidulans* infection associated with a Hickman catheter in a patient with neutropenia. Clin Infect Dis. 1999;29:1594–6. 10.1086/313552 10585834

[6] Dotis J, Roilides E. Osteomyelitis due to *Aspergillus* spp. in patients with chronic granulomatous disease: comparison of *Aspergillus nidulans* and *Aspergillus fumigatus.* Int J Infect Dis. 2004;8:103–10. 10.1016/j.ijid.2003.06.001 14732328

[7] Winkelstein JA, Marino MC, Johnston RB Jr, Boyle J, Curnutte J, Gallin JI, Chronic granulomatous disease. Report on a national registry of 368 patients. Medicine. 2000;79:155–69. 10.1097/00005792-200005000-00003 10844935

[8] Van ‘t Hek LG, Verweij PE, Weemaes CM, Van Dalen R, Yntema JB, Meis JF. Successful treatment with voriconazole of invasive aspergillosis in chronic granulomatous disease. Am J Respir Crit Care Med. 1998;157:1694–6. 9603157 10.1164/ajrccm.157.5.9709068

[9] Berger PE, Warris A, Weemaes CM. Preventing fungal infections in chronic granulomatous disease. N Engl J Med. 2003;349:1190–1. 10.1056/NEJM200309183491220 14503544

[10] Dotis J, Panagopoulou P, Filioti J, Winn R, Toptsis C, Panteliadis C, Femoral osteomyelitis due to *Aspergillus nidulans* in a patient with chronic granulomatous disease. Infection. 2003;31:121–4. 10.1007/s15010-002-2167-1 12682820

[11] Morris A, Schell WA, McDonagh D, Chaffee S, Perfect JR. Pneumonia due to *Fonsecaea pedrosoi* and cerebral abscesses due to *Emericella nidulans* in a bone marrow transplant recipient. Clin Infect Dis. 1995;21:1346–8. 8589181 10.1093/clinids/21.5.1346

[12] Gugnani HC, Vijayan KK, Tyagi P, Sharma S, Stchigel AM, Guarro J. Onychomycosis due to *Emericella quadrilineata.* J Clin Microbiol. 2004;42:914–6. 10.1128/JCM.42.2.914-916.2004 14766889 PMC344492

[13] Henry T, Iwen PC, Hinrichs SH. Identification of *Aspergillus* species using internal transcribed spacer regions 1 and 2. J Clin Microbiol. 2000;38:1510–5. 10747135 10.1128/jcm.38.4.1510-1515.2000 PMC86477

[14] Glass NL, Donaldson GC. Development of primer sets designed for use with the PCR to amplify conserved genes from filamentous ascomycetes. Appl Environ Microbiol. 1995;61:1323–30. 7747954 10.1128/aem.61.4.1323-1330.1995 PMC167388

[15] Samson RA, Houbraken JAMP, Kuijpers AFA, Frank JM, Frisvad JC. New ochratoxin or sclerotium producing species in *Aspergillus* section *Nigri.* Stud Mycol. 2004;50:45–61.

[16] Hong SB, Cho HS, Shin HD, Frisvad JC, Samson RA. Novel *Neosartorya* species isolated from soil in Korea. Int J Syst Evol Microbiol. 2006;56:477–86. 10.1099/ijs.0.63980-0 16449461

[17] Thompson JD, Gibson TJ, Plewniak F, Jeanmougin F, Higgins DG. The ClustalX windows interface: flexible strategies for multiple sequence alignment aided by quality analysis tools. Nucleic Acids Res. 1997;25:4876–8. 10.1093/nar/25.24.4876 9396791 PMC147148

[18] Kimura M. A simple method for estimating evolutionary rates of base substitutions through comparative studies of nucleotide sequences. J Mol Evol. 1980;16:111–20. 10.1007/BF01731581 7463489

[19] Felsenstein J. PHYLIP (Phylogeny inference package), version 3.57c. Seattle: University of Washington; 1995.

[20] Saitou N, Nei M. The neighbor-joining method: a new method for reconstructing phylogenetic trees. Mol Biol Evol. 1987;4:406–25. 3447015 10.1093/oxfordjournals.molbev.a040454

[21] Felsenstein J. Confidence limits on phylogenies: an approach using the bootstrap. Evolution Int J Org Evolution. 1985;39:783–91. 10.2307/2408678 28561359

[22] Swofford T. PAUP*: Phylogenetic analysis using parsimony, version 4.0. Sunderland (MA): Sinauer Associates; 2000.

[23] National Committee for Clinical Laboratory Standards. Reference method for broth dilution antifungal susceptibility testing of filamentous fungi. Approved standard M38-A. Wayne (PA): The Committee; 2002.

[24] Kurtz MB, Heath IB, Marrinan J, Dreikorn S, Onishi J, Douglas C. Morphological effects of lipopeptides against *Aspergillus fumigatus* correlate with activities against (1,3)-D-glucan synthase. Antimicrob Agents Chemother. 1994;38:1480–9. 7979276 10.1128/aac.38.7.1480 PMC284580

[25] Horie Y. Ascospore ornamentation and its application to the taxonomic re-evaluation in *Emericella* [in Japanese]. Transactions of the Mycological Society of Japan. 1980;21:483–93.

[26] Polacheck I, Nagler A, Okon E, Drakos P, Plaskowitz J, Kwon-Chung KJ. *Aspergillus quadrilineatus*, a new causative agent of fungal sinusitis. J Clin Microbiol. 1992;30:3290–3. 1452721 10.1128/jcm.30.12.3290-3293.1992 PMC270654

[27] Drakos PE, Nagler A, Or R, Naparstek E, Kapelushnik J, Engelhard D, Invasive fungal sinusitis in patients undergoing bone marrow transplantation. Bone Marrow Transplant. 1993;12:203–8. 8241977

[28] Ng KP, Saw TL, Madasamy M, Soo Hoo T. Onychomycosis in Malaysia. Mycopathologia. 1999;147:29–32. 10.1023/A:1007044720147 10872513

[29] Tong QJ, Chai WX, Wang ZF, Kou JF, Qi ZT, Wang DL. A case of cerebral aspergillosis caused by *Aspergillus nidulans*. Clinical, pathologic and mycologic identifications. Chin Med J (Engl). 1990;103:518–22. 2119968

[30] Yano S, Kobayashi K, Shishido S, Nakano H. Intrabronchial *Aspergillus nidulans* infection in an immunocompetent man. Intern Med. 1999;38:372–5. 10.2169/internalmedicine.38.372 10361913

[31] Singh MP, Singh CM. Fungi associated with suppurative mycosis of cattle and sheep in India. Indian J Anim Health. 1970;9:432–49.

[32] White CJ, Kwon-Chung KJ, Gallin JI. Chronic granulomatous disease of childhood. An unusual case of infection with *Aspergillus nidulans* var. *echinulatus.* Am J Clin Pathol. 1988;90:312–6. 3046321 10.1093/ajcp/90.3.312

[33] Mitchell RG, Chaplin AJ, Mackenzie DW. *Emericella nidulans* in a maxillary sinus fungal mass. J Med Vet Mycol. 1987;25:339–41. 10.1080/02681218780000401 3323452

[34] Balajee SA, Gribskov J, Brandt M, Ito J, Fothergill A, Marr KA. Mistaken identity: *Neosartorya pseudofischeri* and its anamorph masquerading as *Aspergillus fumigatus.* J Clin Microbiol. 2005;43:5996–9. 10.1128/JCM.43.12.5996-5999.2005 16333088 PMC1317194

[35] Balajee SA, Nickle D, Varga J, Marr KA. Molecular studies reveal frequent misidentification of *Aspergillus fumigatus* by morphotyping. Eukaryot Cell. 2006;5:1705–12. 10.1128/EC.00162-06 17030996 PMC1595351

[36] Kontoyiannis DP, Lewis RE, May GS, Osherov N, Rinaldi MG. *Aspergillus nidulans* is frequently resistant to amphotericin B. Mycoses. 2002;45:406–7. 10.1046/j.1439-0507.2002.00797.x 12421291

[37] Singh J, Rimek D, Kappe R. Intrinsic in vitro susceptibility of primary clinical isolates of *Aspergillus fumigatus, Aspergillus terreus, Aspergillus nidulans, Candida albicans* and *Candida lusitaniae* against amphotericin B. Mycoses. 2006;49:96–103. 10.1111/j.1439-0507.2006.01197.x 16466441

[38] Bowman JC, Abruzzo GK, Flattery AM, Gill CJ, Hickey EJ, Hsu MJ, Efficacy of caspofungin against *Aspergillus flavus, Aspergillus terreus*, and *Aspergillus nidulans.* Antimicrob Agents Chemother. 2006;50:4202–5. 10.1128/AAC.00485-06 17015628 PMC1693977

