# Supplementary material for: Emericella quadrilineata as Cause of Invasive Aspergillosis
Source: Emerg Infect Dis. 2008 Apr;14(4):566–72. doi: 10.3201/eid1404.071157 (PMC2570940; doi:10.3201/eid1404.071157)
Supplement: Appendix Table — Chagas disease cases reported in France since 2004* [file 07-1157_appT.pdf]

Online Appendix Table. Characteristics of 33 *Emericella* isolates identified by morphologic methods and sequence-based analysis*

Original morphologic identification, *Emericella*	Strain no.	Origin*	Disease classification†	Subsequent sequence-based identification, *Emericella*	MIC (mg/L)†					
					AmB	ITZ	VCZ	PCZ	CAS	TBF
nidulans	CBS 589.65^T	G. Bainier, Belgium	–	*nidulans*	4	0.125	0.25	0.063	0.25	0.016
nidulans	CBS 121.35	P. Henrard, unknown	–	*nidulans*	16	0.125	0.5	0.063	0.5	0.031
nidulans	CBS 288.95	M. Klich, USA	–	*nidulans*	4	0.063	0.25	0.063	0.25	0.016
nidulans	CBS 565.70	Cotton yam, UK	–	*nidulans*	4	0.125	0.25	0.063	0.5	0.016
nidulans	AZN9059	Outside air, Oslo, Norway	–	*nidulans*	2	0.063	0.25	0.031	0.25	0.016
nidulans	AZN9043	Outside air, Oslo, Norway	–	*nidulans*	2	0.063	0.25	0.031	0.25	0.016
nidulans	V44-45	Sputum, patient with pulmonary mycobacterial disease and cavitary lung disease, (NIH, USA)	Colonization	*nidulans*	2	0.125	0.25	0.063	0.25	0.016
nidulans	V44-46	Sputum, patient with common variable immunodeficiency and bronchiectasis, (NIH, USA)	Colonization	*nidulans*	4	0.063	0.25	0.063	0.5	0.016
nidulans	V44-48	Sputum, patient with colon cancer, (NIH, USA)	Colonization	*nidulans*	4	0.125	0.25	0.063	0.25	0.016
nidulans	V32-12	BAL-fluid, patient with MDS, (Nijmegen, Netherlands)	Probable IA	*nidulans*	1	0.125	0.25	0.063	0.063	0.031
nidulans	V45-01	Skin swab (Nijmegen, Netherlands)	Colonization	*nidulans*	2	0.063	0.25	0.063	0.25	0.016
nidulans	V06-20	Nose swab (Nijmegen, Netherlands)	Colonization	*nidulans*	2	0.063	0.25	0.063	0.25	0.016
quadrilineata	CBS 591.65^T	Soil, USA	–	*quadrilineata*	0.5	0.125	0.5	0.063	0.5	0.031
nidulans	CBS 291.95	M. Klich, USA	–	*quadrilineata*	0.25	0.125	0.5	0.125	0.5	0.016
nidulans	V42-43	Brain biopsy, BMT recipient (Duke, USA) (1)	Proven cerebral aspergillosis	*quadrilineata*	0.25	0.125	0.25	0.063	4	0.063
nidulans	V44-47	Lung biopsy, patient with CGD (NIH, USA)	Proven IA	*quadrilineata*	1	0.125	0.5	0.063	2	0.016
nidulans	V49-25	Lung biopsy, patient with CGD, (NIH, USA)	Proven IA	*quadrilineata*	0.5	0.063	0.5	0.063	2	0.016
nidulans	V49-18	Sputum, patient with bronchiectasis (Nijmegen, Netherlands)	Colonization	*quadrilineata*	1	0.25	0.5	0.125	2	0.016
nidulans	V24-62	Bronchial aspirate (Nijmegen, Netherlands)	Colonization	*quadrilineata*	0.5	0.125	0.5	0.063	2	0.016
quadrilineata	V40-61	BAL-fluid, patient with CGD (case 1, Nijmegen, Netherlands)	Probable IA	*quadrilineata*	0.25	0.125	0.5	0.125	4	0.016
quadrilineata	V42-23	Bronchial aspirate (case 2, Nijmegen, Netherlands)	Colonization	*quadrilineata*	0.25	0.063	0.25	0.063	4	0.016
quadrilineata	V43-21	Sputum (case 3, Nijmegen, Netherlands)	Colonization	*quadrilineata*	0.5	0.125	0.5	0.063	4	0.016
	V42-81	Brain biopsy, patient with	Proven cerebral	*quadrilineat*	0.5	0.1	0.5	0.0	0.5	0.016

quadrilineata		CLL (case 4, Nijmegen, Netherlands)	aspergillosis	*a*		25		63		
quadrilineata	V43-63	Nail (Bulandshahr, India) (2)	Proven onychomycosis	*quadrilineata*	0.5	0.125	0.25	0.063	0.5	0.016
rugulosa	CBS 198.88	*Amaranthus* sp., USA	–	*rugulosa*	1	0.125	0.5	0.125	0.25	0.031
rugulosa	CBS 171.71	Hay in compost heap, Canada	–	*rugulosa*	1	0.063	0.25	0.063	0.5	0.016
rugulosa	CBS 199.88	*Amaranthus* sp., USA	–	*rugulosa*	1	0.25	0.5	0.125	0.5	0.031
nidulans	V43-77	Bone, patient with CGD (Thessaloniki, Greece) (3)	Proven IA	*rugulosa*	1	0.125	0.25	0.063	1	0.031
nidulans var. *echinulata*	CBS 120.55[T]	Soil, Argentina	–	*nidulans* var. *echinulata*	0.25	0.125	0.25	0.031	2	0.031
nidulans	NCPF 7552	Brain tissue, patient with CGD	Proven cerebral aspergillosis	*nidulans* var. *echinulata*	0.25	0.25	0.25	0.125	4	0.016
nidulans	AZN4606	Biopsy, patient with CGD (Nijmegen, Netherlands) (4)	Proven IA	*nidulans* var. *echinulata*	0.25	0.125	0.5	0.063	4	0.016
nidulans	V53-70	Sputum, patient with COPD (Nijmegen, Netherlands)	Colonization	*nidulans* var. *echinulata*	0.125	0.125	0.5	0.063	2	0.016
heterothallica	CBS 489.65[T]	Soil, Costa Rica	–	*heterothallica*	0.5	0.125	2	0.063	0.5	0.016

*NIH, National Institutes of Health, Bethesda, MD, USA; BAL, bronchoalveolar lavage; MDS, myelodysplastic syndrome; Duke, Duke University, Durham, NC, USA; BMT, bone marrow transplant; CGD, chronic granulomatous disease; CLL, chronic lymphoblastic leukemia. [T]indicates type strain,

†Cases of invasive aspergillosis (IA) were classified according to the EORTC/MSG consensus definitions (5).

‡MICs were determined by using a microbroth dilution format according to the Clinical Laboratory Standards Institute M38-A protocol (6). For caspofungin the minimal effective concentration was determined (7). AmB, amphotericin B; ITZ, itraconazole ; VCZ, voriconazole; PCZ, posaconazole; CAS, caspofungin; TBF, terbinafine.
